# Supplementary material for: Complexome profiling of the Chlamydomonas psb28 mutant reveals TEF5 as an early PSII assembly factor
Source: Plant Cell. 2025 Mar 17;37(6):koaf055. doi: 10.1093/plcell/koaf055 (PMC12164590; doi:10.1093/plcell/koaf055)
Supplement: koaf055_Supplementary_Data [file koaf055_supplementary_data.zip › psb28_interactive.zip!/plots/intensity/qualityPlots/peptides/CPL22_Cre16.g678000.t1.2.html]

### Cre16.g678000.t1.2

Trivial name: CPL22  
SeqCoverage [median]: 14.30  
Mass: 33.09 kDa  
  
 

| Sequence | MissedCleavage | Mass [Da] | wtIntensity | muIntensity | Score |
| --- | --- | --- | --- | --- | --- |
| GANNVVGGMSFATLAR | 0 | 1563.78 | 1027.92 | 3088.55 | 94.19 |
| LYQAVIR | 0 | 861.51 | 871.68 | 0.00 | 106.76 |
| VAYDSEFPIK | 0 | 1167.58 | 0.00 | 3773.13 | 67.90 |
| YQALGGLDAGLVK | 0 | 1303.71 | 1219.13 | 6944.83 | 82.17 |
|  |  |  | 3118.7 | 13806.5 |  |
